# How to Approach a Child About Concerns for Their Mental Health and Seeking Help: A Delphi Expert Consensus Study to Develop Guidelines on Mental Health First Aid for Supporting Children

**DOI:** 10.1111/hex.70126

**Published:** 2025-01-13

**Authors:** Catherine L. Johnson, Claire M. Kelly, Anthony F. Jorm, William Garvey, Laura M. Hart

**Affiliations:** ^1^ Centre for Mental Health and Community Wellbeing, Melbourne School of Population and Global Health, The University of Melbourne Carlton Victoria Australia; ^2^ Centre for Community Child Health The Royal Children's Hospital Melbourne Parkville Victoria Australia; ^3^ Mental Health First Aid International Melbourne Victoria Australia; ^4^ School of Psychology and Public Health La Trobe University Bundoora Victoria Australia

**Keywords:** child mental health, Delphi study, guidelines, mental health first aid

## Abstract

**Background:**

Adults who live or work with children are an important source of support and are gateways to professional help when a child is experiencing a mental health problem. This study aimed to develop consensus‐based guidelines on how adults such as parents, educators or health professionals should approach a child aged 5–12 years to discuss concerns about the child's mental health and seek help.

**Methods:**

A Delphi consensus method with three rounds was used. Experts were recruited from six countries to form three panels: health professionals, educators and people with lived experience (parents and carers, and young people with mental health problems). Statements to be rated were sourced from an online search of websites designed for adults who live or work with children. Further suggestions for statements came from panellists. Statements that reached 80% consensus across all panels were included in the guidelines.

**Results:**

132 participants completed the Round 1 survey, reducing to 54 by Round 3. A total of 248 statements were presented to panel members, with 151 being endorsed and included in the guidelines.

**Conclusions:**

These guidelines represent the first recommendations developed for members of the public providing mental health first aid to children aged 5–12 years.

**Patient or Public Contribution:**

Lived experience advocates (i.e. those with lived experience of a mental health problem in childhood and/or caregiving experience of raising a child with a mental health problem) were involved at two stages of this research: As part of the Advisory Group for the project and as expert panel members. Advisory Group members provided input into the conduct of the study and the content and design of the research outputs. Panel members provided their expertise to review every item to be included in the guidelines, proposed new items to be included, and reviewed and approved the finalised output documents.

**Abbreviations**
MHFASC: Mental Health First Aid for Supporting Children
MHP: mental health problem

People who live and work with children, such as parents, educators and health professionals, are a key source of support for children with mental health problems (MHPs) and can act as gateways to professional help. They may be the first to notice changes in a child's behaviour, emotions or functioning and how they interpret what they observe influences the decision to seek further help for a child [1, 2]. Models of help‐seeking for children, such as the *Gateway Provider Model*, place central importance on the characteristics of parents and other adults in a child's support network as key facilitators of help‐seeking [3]. For example, a parent's knowledge of child MHPs and the parent's skills and confidence in talking to their child about their symptoms can enhance or form a barrier to the help‐seeking process [4, 5]. Reviews of barriers to help‐seeking suggest that a parent's understanding of MHPs in childhood can assist not only the child but also health professionals in recognising and providing appropriate support for MHPs in childhood [6]. Improving the knowledge and skills of parents and adults who work with children is, therefore, likely to support improvements in help‐seeking and, ultimately, treatment access for children with emerging or established MHPs [7]. Indeed, the need for interventions to increase adults' *mental health literacy for supporting children* [7] is increasingly recognised in policy frameworks and government funding initiatives [8, 9].

## 1 Mental Health First Aid for Supporting Children

Mental health first aid for supporting children is defined as ‘*…the help that is given to a child (aged 5‐12 years), or to the caregiving adults of a child who: (1) is developing a mental health problem or is experiencing a worsening of an existing problem, (2) has experienced an adversity or traumatic event that increases risk of poor mental health, or (3) is in a mental health crisis. The first aid is given to the child or their primary support system until appropriate help is received*’ [10]. The mental health first aid model emphasises the role of the public in providing appropriate support to children developing or experiencing MHPs. Mental health first aid (MHFA) was originally developed as a model for adults to support other adults with mental health problems [11]. The not‐for‐profit training organisation, Mental Health First Aid International (MHFAI), offers training courses in how to provide mental health first aid to other adults. This original course emphasises assessing, listening, giving reassurance and information, encouraging professional help, and encouraging other supports. Although there is both a *Youth MHFA* version of the course (for adults providing mental health first aid to a young person) and a *Teen MHFA* course (for adolescents providing first aid to a peer), neither of these courses has specific guidance that considers the unique developmental needs of children [12, 13].

Large scale research on how the public views childhood MHPs, such as the *National Stigma Study‐Children* in the USA and the FrameWork Institute's review of the scientific and public child mental health discourse [14, 15, 16] has been undertaken to determine how child mental health may be conceptualised and understood. However, there has been little research on the specific knowledge and skills required by parents, educators and other adults who live or work with children, such as health professionals. A much greater literature focuses on how adults who live and work with adolescents can support youth mental health; as found by [17] in their review of parents' mental health literacy.

Many countries have now enacted laws making it mandatory for adults who live or work with children to report concerns or evidence of a child experiencing abuse or neglect [18, 19]. While the scope and nature of these laws can vary widely from one country or jurisdiction to another, a common implication is the need for adults who support children to have knowledge and skills in understanding how to speak with a child about potential symptoms of concern. While many workplaces have implemented compulsory training for their employees on how to adhere to the legislative requirements of mandatory reporting of child neglect or abuse, there is a distinct lack of interventions that provide guidance for adults on how to speak with a child in a way that protects and supports the child's mental health [20]. These knowledge and skills appear to be needed across even those professions who are mandatory reporters. For example, there is evidence that even with their professional training, general health care professionals (e.g. general practitioners or primary care physicians) report a lack of confidence and skills in meeting the needs of children with MHPs [21, 22, 23].

The Delphi method has been used extensively in health research to develop best‐practice guidelines where there is insufficient evidence available from trials or observational studies, or the generation of such evidence is not feasible or timely [24, 25]. The Delphi method relies on gathering consensus from panels of experts who have relevant knowledge and experience, either through their professional work or through lived experience, and has the benefit of avoiding some of the pitfalls of group decision‐making, such as conformity and group‐think [26]. The method is supported by ‘wisdom of crowds’ research showing that groups can make good judgements under certain conditions [27]. The Delphi method has been used to develop a range of other relevant guidelines, such as those for providing mental health first aid to adolescents [28, 29].

Given the clear gap that exists for guidance on how adults could support a child experiencing a mental health problem, the aim of this study was to use expert consensus to determine the most safe and feasible actions an adult could take in responding to a child who they suspect may have an established or emerging mental health problem. These approaches could then be used in interventions worldwide that are designed to improve mental health literacy, knowledge and skills. The study was part of a larger research project to develop the basis for developing a training program on *Mental Health First Aid for Supporting Children*. Before commencing this study, the research team conducted a needs assessment with educators, parents and health professionals around Australia to find out about what their needs were in regard to understanding MHPs in children and supporting children who experience MHPs. From this needs assessment, a conceptual model was developed by the first and last authors that identified four areas of need: (1) talking to a child about concerns for their mental health, (2) involving other adults in the child's support system in conversations about a child's mental health, (3) how to help a child who is experiencing a mental health crisis and (4) how to help a child who has experienced an adverse event (trauma). This article describes the development of guidelines for the first of these four topics.

## 2 Method

Strategies that an adult could use when talking to a child about their mental health were developed via a search of the literature, development of a questionnaire and three Delphi consensus rounds. Criteria for consensus between panels were determined a priori according to previously published Delphi studies in mental health research [29]. This study was not registered a priori and is reported according to the Accurate Consensus Reporting Document (ACCORD) guidelines [30].

### 2.1 Literature Searches

A Google search across Google Australia, US, UK, Canada and New Zealand was developed using key search terms, which were adapted and updated from previous Delphi studies on adolescents [29]. The search terms were ‘How to help’ OR talk OR communicate OR discuss AND mental health problem/depression/anxiety/eating disorders/ADHD/autism AND child* OR kid OR primary/elementary student, as well as colloquial phrases like ‘How do I help a child with depression?’ The first 50 websites from each search engine were extracted into an Excel file, and duplicate websites were removed. Websites were then screened for information about knowledge or actions an adult could take to approach and have a supportive discussion with a child aged 5–12 years old who they suspect is experiencing or developing a mental health problem. Information was extracted and drafted into mental health first aid for supporting children knowledge or action statements. Any relevant reports, brochures or marketing material that were hosted on the included websites were also downloaded and screened for relevant statements. Another search of Google Books and Amazon Books was searched using the same search terms, and any relevant books were located and relevant statements extracted. A search of the academic literature was also performed via PsycINFO, MEDLINE and Social Science databases with the key search terms: ((mental health problem OR disorder OR concern OR illness) AND children AND (help OR assistance OR intervene OR support)). Full search terms and further information can be found in Supporting Information S1: File A. All searches were conducted between April and July of 2021.

### 2.2 Questionnaire Development

Statements identified across the searches were first categorised into broad themes by the first author. Three broad themes were identified: knowledge or actions relevant to adults preparing for a conversation with a child, knowledge or actions relevant to an adult during a conversation with a child and knowledge or actions relevant to an adult after the conversation had taken place. Within each of these three broad categories, statements that contained similar ideas were grouped together. The first author then redrafted the statements so that they each contained only one main idea and were phrased in a way that made them actionable (i.e. contained an action an adult could take or a piece of knowledge they should be aware of). A working group consisting of researchers with previous experience in child mental health, MHFA training and/or Delphi studies then reviewed all statements to ensure they were within the scope of mental health first aid for supporting children and redrafted them as necessary. When classifying statements into themes, it emerged that some statements had particular relevance to groups of people, such as parents, educators or health professionals. These statements were therefore clustered as specific knowledge or actions that these different supporters might use.

Some statements related to actions an adult could take to engage in a conversation with a child who is experiencing a mental health crisis, which involves an increased risk of harm, either to the child or others around them. Providing mental health first aid under these circumstances was outside the scope of the current study. However, any statements uncovered in the search that were relevant to crises were entered into a separate, subsequent study focused specifically on how adults can support children experiencing a mental health crisis.

### 2.3 Recruitment

Oversight of the study (including research conduct, design and validation of research outputs) was provided by both the working group and an Advisory Group consisting of health professionals, parents and carers with lived experience of raising a child with MHPs, educators, policy experts and researchers with experience in Delphi studies and MHFA. The Advisory Group met quarterly over the course of a year before the data collection phase of this project.

We recruited four panels with specific expertise (professional or lived experience) in child mental health through both professional and personal networks, as well as social media posts, consumer advocacy groups and peak professional bodies. Participants were recruited from countries where MHFA has an established organization, such as Australia, USA, England, Ireland, Germany and New Zealand.

The four panels were as follows:

1. Mental health professionals who has been providing mental health care/treatment to children aged 5–12 years for 5 years or more
2. Educators, school staff or school‐based health professionals working with primary/elementary school students with at least 5 years of experience working with children with MHPs or crises
3. Young people aged between 16 and 25 years, who had experienced an MHP in primary/elementary school, felt well enough to participate and were engaged in advocacy activities (i.e. a member of an advisory or advocacy group, or providing peer support to others)
4. Parent/carer/guardians of a child who experienced/is experiencing an MHP at any time between 5 and 12 years of age and were engaged in advocacy activities (i.e. a member of an advisory or advocacy group, or providing peer support to others)

Participants received a flyer via email or social media post, where they could scan a Quick Response (QR) code or click a link to be taken to an enrolment survey. Participants were encouraged to pass on the link to any other interested parties in their networks. The enrolment survey incorporated a Plain Language Statement explaining the purposes and aims of the study, an eligibility check that asked participants to provide details on their expertise in child mental health and some demographic details, and an active consent check. Lived experience panel members were compensated with a gift card for $50 AUD if they completed all three rounds of the survey. The first author reviewed the eligibility checks and if necessary, contacted participants directly to confirm their expertise and suitability for inclusion on the Delphi panels.

### 2.4 Delphi Rounds

The statements were developed into a questionnaire for presentation to the expert panellists. Statements were presented to participants via an online survey platform (Qualtrics) to allow panellists to access the survey at a time that suited them. Panel members were asked to individually rate how important each action or knowledge statement was to providing safe and effective mental health first aid to a child during a conversation, using the following options: *Essential, Important, Don't Know/Depends, Unimportant, Should Not Be Included*.

In Round 1, panel members were also invited to make comments on any ambiguity in the statements presented and to suggest any new ones that had not yet been considered. Panel members were also asked if they would rate any of the statements differently if the first aider was a parent, a health professional or a teacher, if the statements were in relation to an older child (defined as 9–12 years old) or a younger child (5–8 years old) or if there was a particular mental health problem present. The panel members provided over 600 comments. Comments that represented a new idea that had not yet been included were drafted into statements by the first author and presented to the working group, where a final decision was made about their format and presentation for Round 2.

Following Round 1, panel members received a report of their rating of each item, a list of all endorsed and rejected items, and an anonymous overview of how all the panels had rated each item that would be re‐rated in Round 2. This allowed panel members to compare their responses to the other panels and decide whether they would change their response in Round 2 or keep it the same.

### 2.5 Data Analysis

A low number of young people completed Round 1 of the survey, thus the parents and the young people were combined into a ‘lived experience panel’ for analysis, which consisted of 33 members (2 young people + 31 parents). Ratings of all items was therefore completed by three panels: health professionals, educators and lived experience experts.

### 2.6 Criteria for Consensus

In Round 1, a statement was ‘endorsed’ if at least 80% of all three panels rated the item as either ‘Essential’ or ‘Important’. Statements were re‐rated in Round 2 if: (a) 70%–79% of all three panels rated an item as ‘Essential’ or ‘Important’, or (b) it was rated ‘Essential or Important’ by at least 80% of one panel, but the other panels did not reach consensus (under 80% rating it as Essential or Important). A statement was rejected if less than 70% of at least one panel rated it as ‘Essential’ or ‘Important’ and no panel rated it at 80% or above [27, 29, 31, 32, 33, 34, 35].

The same criteria were applied to the data collected in Rounds 2 and 3, except that if a statement was re‐rated in the second round and again failed to achieve a consensus of 80%–100% across all three panels, it was then excluded. Only those statements that had been entered as new items in Round 2, and fell into the *Re‐rate* category, were included in the Round 3 survey.

### 2.7 Guideline Development

The first author grouped endorsed statements together into a cohesive sequence and under broad sections (such as approaching the child, what to do if they do not want to talk to you), and then drafted these sections into prose. The guidelines were then presented to the working group, which edited the document to flow more cohesively, whilst keeping the original meaning of each of the statements. The guidelines document was sent to panel members who completed all three rounds of the Delphi survey for endorsement as a set of finalised guidelines (see Supporting Information S2: File B).

## 3 Results

### 3.1 Participants

Round 1 was completed by 132 (9 males and 123 females) participants, comprising 48 health professionals (psychologists, paediatricians, mental health nurses, occupational therapists, psychiatrists) (*MAge* = 45.59 years), 31 parents or carers, 2 young people with lived experience of MHPs in childhood and 51 educators of primary or elementary aged children (*MAge* = 46.22 years). The panelists were from Australia (36), USA (87), New Zealand (5) and other countries (including Germany, Switzerland, Ireland – 4). Round 1 was held in January 2022, Round 2 in March 2022 and Round 3 in May 2022. Each survey remained open for 2–3 weeks.

Overall, 56.1% of participants who completed Round 1 completed Round 2, and 40.91% of participants completed Round 3. Participant numbers for all three panels remained above the range of generally reported minimum number required for stability of results [36]. An overview of participant completion rates across rounds can be found in Table 1.

**Table 1:** Delphi study participant completion of rounds.

	Total	Health Professionals	Parents	Young People	Educators
Number of enrolled participants	201	58	54	6	83
Number completing Round 1	132	48	31	2	51
%	65.7	82.8	57.4	33.3	61.4
Number completing Round 2	74	26	17	0	31
%	56.1	54.2	54.8	0.0	60.8
Number completing Round 3	54	22	12	0	20
%	40.9	45.8	38.7	0.0	39.2

### 3.2 Results From Rounds

**Figure 1:**
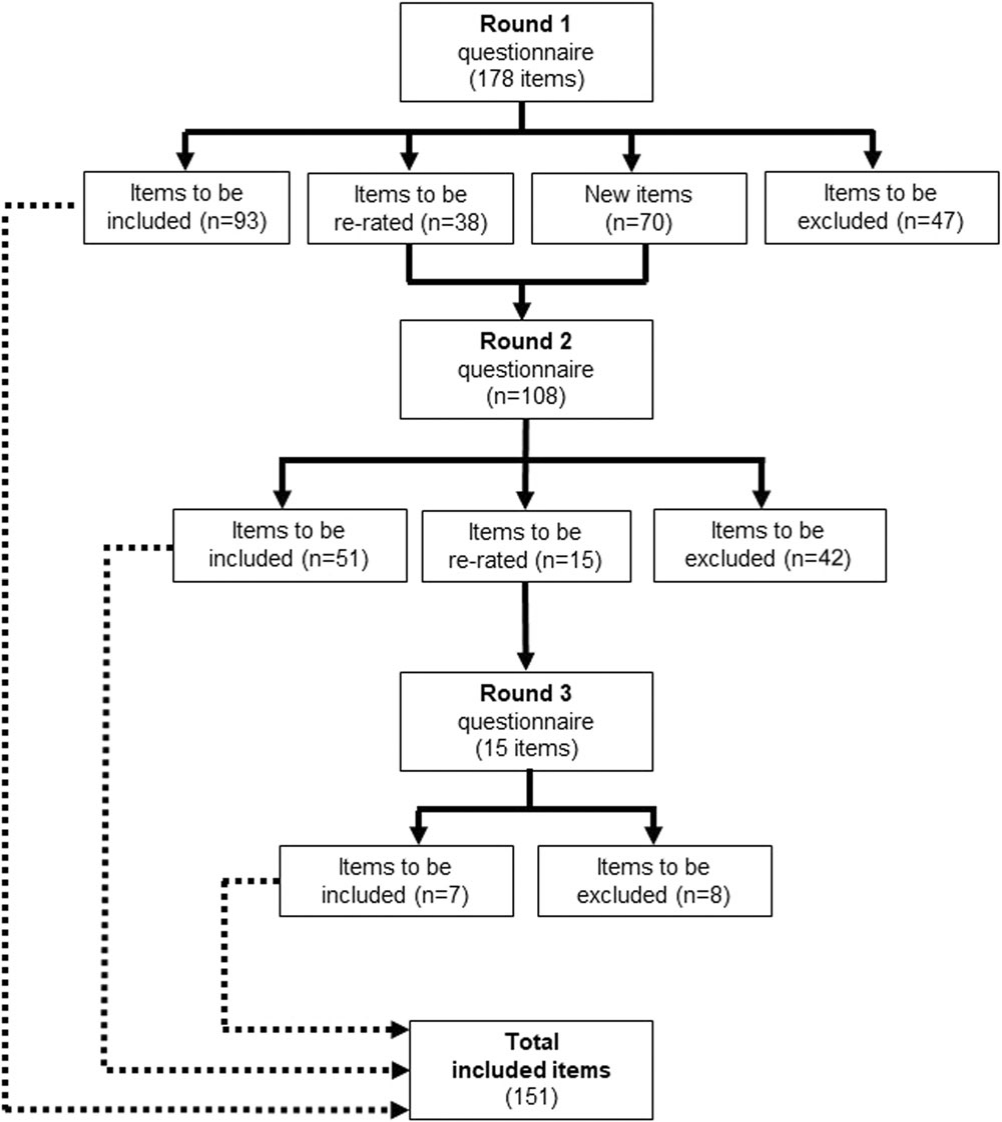
Flow of items through the Delphi rounds.

An overview of all three Rounds is provided in Figure 1. Overall, 187 statements were presented to panel members in Round 1, of which 151 statements were endorsed as guidelines (see Table 2). From the comments provided in Round 1, 61 new statements were developed and entered into Round 2. Statements that were rejected strongly (i.e. more than 40% of the members from all three panels rated the statement as ‘unimportant’ or ‘should not be included’) and those that were contentious (i.e. high variance in the ratings given to the statements between panels) are also presented in Tables 3 and 4.

**Table 2:** Endorsed Delphi statements by round.

Endorsed statement	Round endorsed
Early in the conversation, the first aider should encourage the child to discuss any concerns they have e.g. ‘Is there anything you wanted to talk about? You haven't really seemed like yourself lately’.	1
Early in the conversation, the first aider should let the child know that they are there to listen to the child.	1
Early in the conversation, the first aider should not immediately jump into trying to solve any problem the child may have.	1
Early in the conversation, the first aider should talk about what they have noticed that makes them concerned about the child.	1
If the child does not want to talk, the first aider should tell the child that the first aider is here to talk if the child would like to.	1
If the child expresses myths or misconceptions about mental health problems, the first aider should try to correct these e.g. boys should not cry, girls should not get angry.	1
If the child has an emotional reaction to the conversation, the first aider should acknowledge this and use accurate emotional language e.g. angry, sad, scared.	1
If the child is anxious, the first aider should use a kind and calm communication manner.	1
If the child is reluctant for the first aider to talk to another adult, the first aider should explain why they have to talk to another adult.	1
If the child is reluctant for the first aider to talk to another adult, the first aider should give the child time to think about who the child would like to have help from.	1
If the child is reluctant to seek help, the first aider should ask the child about their worries and concerns.	1
If the child is resisting talking to the first aider, the first aider should ask the child if there is another trusted adult they would be willing to talk to.	1
If the first aider does not know the answer to a question the child asks, the first aider should be honest and tell the child they do not know but will find out the answer.	1
If the first aider is not the child's parent, the first aider should talk to the child about how they would like their parents to support the child.	1
In addition to listening to the child's words, the first aider should also observe the child's tone of voice and body language.	1
The first aider should advocate for the child to receive appropriate professional help as soon as possible.	1
The first aider should answer the child's questions honestly and directly.	1
The first aider should answer the child's questions, taking into account the age, maturity and needs of the child.	1
The first aider should ask follow‐up questions of the child to show their understanding.	1
The first aider should attempt to remain non‐judgmental.	1
The first aider should avoid expressing a negative judgement about the child or their situation.	1
The first aider should avoid shaming, criticising or blaming the child.	1
The first aider should be aware of common mental health problems diagnosed in childhood.	1
The first aider should be aware of evidence‐based programs and resources for adults to help support the child's mental health e.g. online training programs for parents.	1
The first aider should be aware of professionals that can make diagnoses and treat mental health problems in children.	1
The first aider should be aware of risk factors for mental health problems in children.	1
The first aider should be aware of the range of appropriate resources that are available to help adults who support children with mental health problems.	1
The first aider should be aware of the warning signs of mental health problems in children.	1
The first aider should be aware that a fear of being judged or treated differently can stop the child from opening up during a conversation about their mental health.	1
The first aider should be aware that appropriate help for a child with a mental health problem should ideally involve parents and teachers working together with the support of health professionals if further skills or intervention are required.	1
The first aider should be aware that appropriate help for a child with a mental health problem should ideally involve parents, teachers and health professionals working together to support the child.	1
The first aider should be aware that children differ in their cognitive and emotional development and that the first aider needs to tailor their approach, language and actions accordingly.	1
The first aider should be aware that some children may have difficulty communicating about their mental health, especially if they have additional needs or disabilities, and the first aider should offer these children more time and support when discussing mental health.	1
The first aider should be aware that the child might prefer to talk and be heard, rather than focus on problem solving.	1
The first aider should be aware that the earlier a child receives appropriate professional help, the better the outcome is likely to be.	1
The first aider should be aware that the more knowledge they have about child mental health, the more confident they may feel in approaching the child.	1
The first aider should be aware that they may need to have more than one conversation about mental health with the child.	1
The first aider should be aware that when children are struggling with their emotions, they may find it harder than usual to communicate how they are feeling.	1
The first aider should be guided by the child's reactions or responses in determining the direction and pace of the conversation.	1
The first aider should be guided by the child's reactions or responses when making a decision whether to continue the conversation, move onto another topic, or give the child some space to settle.	1
The first aider should be mindful of any negative attitudes they have towards mental illness, as the child may pick up on this.	1
The first aider should choose a time and place to talk with the child where they both feel comfortable, and nobody will disturb them.	1
The first aider should consider seeking professional help for the child if there are symptoms that are having a major impact on child's life (sleeping, eating, schooling, peer relationships, family relationships).	1
The first aider should consider seeking professional help for the child, if there are symptoms that last longer than 4 weeks.	1
The first aider should consider seeking professional help for the child, if there are symptoms that last longer than 2 weeks.	1
The first aider should consider seeking professional help for the child, if there is a risk of harm to child.	1
The first aider should consider seeking professional help for the child, if there is a risk of harm to others.	1
The first aider should consider seeking professional help for the child, if there is high levels of distress in the child.	1
The first aider should consider whether the child might find it easier to talk while doing another activity e.g. playing a game, drawing, kicking a ball.	1
The first aider should consider who the best person is to provide mental health first aid to the child.	1
The first aider should encourage the child to ask questions whenever they want.	1
The first aider should encourage the child to talk to other trusted adults about their problem.	1
The first aider should explain there are ways to deal with mental health problems.	1
The first aider should explain to the child that because of the concerns they have, the first aider is going to talk to someone who can help.	1
The first aider should give the child their full attention.	1
The first aider should give the child time to think and ask questions.	1
The first aider should have information about helplines and online chat services for the child, in case they are needed.	1
The first aider should never promise to keep the conversation a secret.	1
The first aider should not attempt to diagnose a child's mental health problem.	1
The first aider should not ignore, minimise or make fun of a child's fears, even if they appear trivial to the first aider.	1
The first aider should not make assumptions about what is causing a child's mental health problem or what is happening in the child's life.	1
The first aider should not use words or language that minimises or invalidates the child's problem e.g. Do not label the child as ‘silly’ or ‘attention‐seeking’.	1
The first aider should offer the child the option of talking to other trusted adults about their problem.	1
The first aider should offer to revisit the plan if the child's situation changes or if the child wishes to reengage with the conversation.	1
The first aider should only give the child simple and relevant information that they can understand.	1
The first aider should praise the child for talking about their feelings.	1
The first aider should reassure the child that expressing feelings is okay.	1
The first aider should reassure the child that it is ok to talk to a trusted adult about what is concerning them.	1
The first aider should reassure the child that the first aider won't get angry or upset with them.	1
The first aider should reassure the child that there is help available.	1
The first aider should reassure the child that they are not to blame for any feelings they might be having.	1
The first aider should reassure the child that they can tell the first aider anything they feel comfortable talking about.	1
The first aider should reassure the child that they will take the time to answer any questions they have.	1
The first aider should remember that children may not always have the language to communicate how they feel.	1
The first aider should show the child that they are actively listening e.g. by using reflective statements.	1
The first aider should slow down or pause the conversation if the child becomes distressed or disengaged.	1
The first aider should take the child's concerns seriously, even if it does not feel like a big deal to them.	1
The first aider should tell the child that they care.	1
The first aider should try not to react too strongly to what the child has to say.	1
The first aider should try not to take it personally if the child does not open up straight away.	1
The first aider should try to accept the child's feelings, even if they are different to the first aider's feelings.	1
The first aider should try to avoid arguments.	1
The first aider should try to create an emotionally safe place where the child can talk openly.	1
The first aider should try to keep their body language open and relaxed.	1
The first aider should try to put themselves in the child's shoes and show empathy for their situation.	1
The first aider should try to sit down at the child's level.	1
The first aider should try to stay calm when speaking with the child.	1
The first aider should try to think about the situation from the child's point of view.	1
The first aider should use language and communication strategies that are appropriate to the child's age or developmental level.	1
The first aider should use open questions to encourage the child to talk.	1
When providing mental health first aid, the first aider should consider the age, maturity and needs of the child.	1
When speaking with a child about mental health problems, the first aider should avoid using negative or stigmatising language e.g. weak, crazy, naughty.	1
When starting the conversation, the first aider should explain there are limits to what can be kept confidential (e.g. ‘kept between us’).	1
Adults providing mental health first aid to a child should have a basic understanding of how mental health problems may present in children with neurodevelopmental disorders.	2
During the conversation, the first aider should let the child know it is OK to ask for a break if they need one.	2
Early in the conversation, the first aider should try to build trust and rapport with the child.	2
If age/developmentally appropriate, the first aider should try to involve the child in any decisions about when and where they have the conversation.	2
If age/developmentally appropriate, the first aider should try to work with the child to decide who else should be informed.	2
If the child does not like to talk, the first aider should use other ways to help them communicate (e.g. notes, drawing, texting).	2
If the child is describing physical symptoms (i.e. stomach ache, head ache), the first aider should explain how sometimes thoughts and feelings can lead to physical symptoms.	2
If the child is having difficulty communicating, or does not like to talk, the first aider should consider using alternative means of communicating e.g. drawing, modelling or playing with toys.	2
If the child is not willing to talk, the first aider should seek support from other adults who could help the child.	2
If the child reports any of the following, the First Aider should follow the crisis First Aid guidelines – Abuse or trauma.	2
If the child reports any of the following, the First Aider should follow the crisis First Aid guidelines *–* Expressing a wish to die.	2
If the child reports any of the following, the First Aider should follow the crisis First Aid guidelines – Self‐harm.	2
If the child reports any of the following, the First Aider should follow the crisis First Aid guidelines – Thoughts or feelings of suicide.	2
If the first aider can't figure out what to do next in helping the child, the first aider should seek help from another trusted adult who could provide a new perspective on the child's problem – (NOTE: Endorsed for an older child, approx. 9–12 years old).	2
If the first aider can't figure out what to do next in helping the child, the first aider should seek help from another trusted adult who could provide a new perspective on the child's problem – (NOTE: Endorsed for a younger child, approx. 5–8 years old).	2
If the first aider is a teacher and has ongoing concerns about the child or is worried about the child's reaction to the conversation, the first aider should seek advice from a mental health professional or an appropriate school staff member with well‐being responsibility.	2
If the first aider is from a different culture to the child, they should try to find out about how mental health problems are understood in the child's culture.	2
If the first aider is not the parent, the first aider should try to support the parents to provide first aid to the child, unless doing so would put the child at risk.	2
The first aider should ask the child if they would like to have another adult present.	2
The first aider should be careful NOT to promise the child that everything will be fine.	2
The first aider should be clear with the child about who they will tell any information that the child provides.	2
The first aider should consider the child's mental health problems within the context of the child's family, school and community.	2
The first aider should discuss with the child who the child feels most comfortable talking to about their problems.	2
The first aider should encourage other adults who care for the child to use evidence‐based strategies (e.g. good sleep habits, regular physical activity, relaxation techniques) to support the child's mental health.	2
The first aider should encourage the child to try to use coping strategies that are evidence‐based (e.g. relaxation techniques) whilst waiting for professional help.	2
The first aider should explain that it is common to experience problems with mental health.	2
The first aider should explain the limits of their confidentiality with the child by discussing when and why other adults may need to be informed.	2
The first aider should explain to the child that the child needs help and the first aider will not ignore or avoid the problem – (NOTE: This statement was endorsed for first aiders who are also parents).	2
The first aider should have an understanding of the impact of trauma on mental health in general.	2
The first aider should have some knowledge about referral pathways and local services available for children.	2
The first aider should have some understanding of protective factors for good child mental health and draw on these when providing first aid.	2
The first aider should have sufficient knowledge about normal child development and behaviours to be able to recognise when a child is developing a mental health problem.	2
The first aider should pause regularly when delivering new information to allow the child time to process.	2
The first aider should reassure the child that they are not going to get in trouble with the first aider for talking about how they feel.	2
The first aider should recommend good‐quality programs and resources (e.g. online programs for parents) for other adults in the child's life, if they are not aware of them. – (NOTE: This statement was endorsed for first aiders who are also parents).	2
The first aider should recommend good quality programs and resources (e.g. online programs for parents) for other adults in the child's life, if they are not aware of them. – (NOTE: This statement was endorsed for first aiders who are also parents).	2
The first aider should recommend good quality programs and resources (e.g. online programs for parents) to other adults in the child's life if they are not aware of them.	2
The first aider should try to avoid confrontation or hostility.	2
The first aider should try to enlist the help of parents when supporting the child, unless doing so would put the child at risk.	2
The first aider should try to talk to the child as soon as possible, but not at the expense of ensuring a comfortable situation for the conversation.	2
The first aider should try to work with the child to come up with a plan together for what to do next – (NOTE: Endorsed for an older child, approx. 9–12 years old).	2
The first aider should try to work with the child to come up with a plan together for who else should be involved – (NOTE: Endorsed for an older child, approx. 9–12 years old).	2
The first aider should use a warm and caring communication style.	2
Throughout the conversation, the first aider should check that the child has understood what has been said e.g. by asking the child to reflect back to the first aider in the child's own words what they understand.	2
Throughout the conversation, the first aider should check that the child has understood what has been said in an age/developmentally appropriate way.	2
To find out what strategies may be useful for the child's self‐care, the first aider should ask the child what makes them feel happy or what they enjoy.	2
When building rapport, the first aider should be guided by the child's interests, skills and strengths.	2
When discussing confidentiality, the first aider should check that the child has understood what has been said e.g. by asking the child to reflect back to the first aider in the child's own words what they understand.	2
When discussing confidentiality, the first aider should check that the child has understood what has been said in an age/developmentally appropriate way.	2
When providing mental health first aid, the first aider should consider the warning signs and risk factors relevant to the child e.g. history of trauma.	2
When speaking with the child, the first aider should be careful to show respect for the child's family.	2
If the child is concerned about the process of seeking help, the first aider should look at appropriate resources with the child e.g. picture books, short videos about going to the doctor/counsellor – (NOTE: This statement was endorsed for first aiders who are also parents). (	3
The first aider should ask the child if they would prefer to have some time talking to the professional alone – (NOTE: Endorsed for an older child, approx. 9–12 years old).	3
The first aider should explain to the child that the child needs help and the first aider will not ignore or avoid the problem – (NOTE: This statement was endorsed for first aiders who are also health professionals).	3
The first aider should recommend good quality programs and resources (e.g. online programs for parents) for other adults in the child's life if they are not aware of them. – (NOTE: This statement was endorsed for first aiders who are also health professionals).	3
The first aider should try to identify one or two adults in the child's life who can keep an eye on how the child is going e.g. a parent may ask a teacher, or a health professional may ask a parent.	3
When talking about mental health problems with the child, the first aider should consider explaining emotional feelings by describing physical sensations in an age/developmentally appropriate way – (NOTE: Endorsed for an older child, approx. 9–12 years old).	3
When talking about mental health problems with the child, the first aider should consider explaining emotional feelings by describing physical sensations in an age/developmentally appropriate way – (NOTE: Endorsed for a younger child, approx. 5−8 years old).	3

**Table 3:** Strongly rejected statements.

Statement	Percentage of panelists rating statements as ‘Unimportant' or ‘Should not be included’		
	Health professionals	Lived experience	Educators
If the child is anxious, the first aider should use a firm and directive communication manner	51.2%	44.8%	66.7%
The first aider should consider normalising the child's mental health problems by talking about the first aider's current struggles with mental health	70.7%	75.9%	76.7%
The first aider should consider normalising the child's mental health problems by talking about their own challenges in life	56.1%	55.2%	60.4%
The first aider should consider normalising the child's mental health problems by referring to the first aider's own experiences of mental health problems or others known to them	53.7%	51.7%	62.8%

**Table 4:** Contentious statements.

Statement	Percentage of panelists rating statements as ‘Essential or Important’		
	Health Professionals	Lived Experience	Educators
The first aider should explain that it is common to experience problems with mental health.	65.9%	96.6%	72.1%
If the child seems uncomfortable with any questions the first aider asks, the first aider should explain why they are asking them and why it is important to talk.	70%	86.2%	61%
If the child seems uncomfortable, the first aider should move off the topic but try to remain engaged with the child and when the child seems more settled, the first aider should try and approach the topic again.	70%	82.8%	68.3%
The first aider should ask other adults close to the child to monitor the child's mental health and keep them informed of any concerns.	82.1%	65.5%	56.1%
The first aider should explain to the child that they need to help the child and will not ignore or avoid the problem.	79.5%	89.7%	65.9%
The first aider should ask the child if they would prefer to have some time talking to a mental health professional alone.	71.8%	93.1%	63.4%

### 3.3 Guidelines Document

The 151 statements were organised into five subheadings, as follows:

1. Background knowledge that a mental health first aider should have (*How to recognise mental health problems, Risk and protective factors, Services available)*. This section focuses on the specific knowledge that a first aider might need when preparing to have a supportive conversation with children.
2. Preparing to give mental health first aid (*Tailor your approach to the child, Consider the best way to communicate with the child, Consider who could help, Find a time and place, Consider the child's family and community context, Consider your own view of mental health*). This section focuses on taking the child's family and community context and developmental stage into account when thinking about how to approach them and communicate with them. In addition, first aiders are encouraged to be mindful of their own attitude towards child mental health and to create a time and place where the child will be most comfortable, including the child in this decision where possible.
3. Talking to the child about their mental health (*Create a supportive atmosphere, Confidentiality, Talk about your concerns, Listen actively, Think about your body language and tone of voice, Resist the urge to problem‐solve, Encourage questions, Take their concerns seriously, Empathise, Explain mental health, Avoid reacting negatively)*. This section focuses on creating a supportive setting for the child, issues of confidentiality and raising concerns with the child, as well as tips for maintaining open body language, empathy, taking concerns seriously and listening skills.
4. Handling difficulties in the conversations (*If the child is reluctant to talk to you, If the child has a strong emotional reaction to the conversation, If you are unsure of what to do next)*. This section focuses on what to do if the first aider faces barriers in the conversation, such as the child being reluctant to talk to them or if there are strong emotional reactions to the conversation. It also covers considering alternative methods of communication (such as playing with toys or drawing) or conversations in parallel to an activity or task (such as kicking a ball).
5. Seeking Help (*Encourage early help, How do you know the child needs professional help?, What is appropriate help for a child?, If the child is reluctant to get others involved, If the child is at risk of harm, Encourage coping strategies)*. This section focuses on encouraging early help, the signs to determine if a child needs professional help, what exactly constitutes appropriate help and how to encourage good coping strategies while waiting for professional help, both for the child and for their support system. The guidelines clearly state that the first aider has a responsibility for seeking help on behalf of the child, in tandem with other responsible and significant adults in the child's life. They also emphasise getting the child's parent/s involved, if it is appropriate and unlikely to harm the child.

## 4 Discussion

A lack of training for health professionals, educators and parents about child mental health has been noted [22, 37], and poor mental health literacy for supporting child mental health can perpetuate stigma [38] and be a barrier to help‐seeking for the child. Thus, programs designed to improve adults' knowledge and skills in approaching a child to have a supportive conversation about mental health and the help‐seeking process, is a key area of need in population mental health. However, exactly how an adult should conduct a conversation with a child about concerns for the child's mental health has remained unclear. Thus, this study aimed to develop practical guidance based on expert consensus for parents, educators or health professionals.

This study identified 151 statements that expert panellists reached a consensus on as being essential or important to the goals of mental health first aid for supporting children. Overall, many of the statements endorsed in this study were similar to ones that have been endorsed in guidelines on providing MHFA to adolescents, particularly on the themes of effective communication and listening empathetically [28] However, in contrast to other guidelines, the statements endorsed in this study contain many recommendations around tailoring the first aider's approach according to the developmental stages and unique needs of children. For example, paying attention to the context of the child, particularly their family context, was a theme in the section ‘Preparing to give mental health first aid’. This is in keeping with a bioecological approach to understanding children's development, which places importance on the children's immediate family, home and school environment in shaping their health and development [39]. Another novel recommendation from this study was for the first aider to ensure that any mental health information is discussed in a way that could be easily comprehended by the child. Other researchers have noted that mental health information and interventions should be delivered to children in a way that is relevant and developmentally appropriate to effectively meet their needs [40]. In light of this, these guidelines include some statements that were endorsed only for older children (aged approximately 9‐12 years old), such as allowing them more autonomy in helping to give input into a plan of action for how to seek help, and who they might like to have involved.

These child‐focused guidelines also include a range of knowledge that would help adults in the recognition of childhood MHPs, the reduction of stigma towards children with MHPs and understanding of the help‐seeking process, all of which have been identified as key for the improvement of a public health response to MHPs in children [41]. These key pieces of knowledge have also been identified as facilitators for help‐seeking by parents [6]. There was a strong endorsement that first aiders' need knowledge of the warning signs of MHPs in children, which are often different to those in adults and adolescents [7]. Knowledge of child development is important when providing first aid to children, particularly in helping the first aider to make a judgement about when to first provide support. This is important because child MHPs are often poorly recognised, perhaps due to their different presentation to that of adolescents and adults, and uncertainty for carers and clinicians around what may be developmentally normal (e.g. developmentally normative fears in young children) and what may be considered pathological (e.g. an anxiety disorder) [7, 9]. Additionally, identification of a child's mental health status as differing from their norm is a fraught task for many parents and can come with associated affiliate stigma [42, 43].

There was strong endorsement (e.g. 100% endorsement from all three panels) that the first aider should avoid the use of stigmatising language, shaming, judging, criticising or minimising the child's concerns. This is in agreeance with other studies that have found that the use of stigmatising language perpetuates negative stereotypes of those with MHPs, and this may be more pertinent in younger children because their cognitive processing is more concrete when assessing others [44]. It is likely, given what we know about the impact of MHFA training and stigma reduction in other populations, that a training model based on these guidelines could help to potentially reduce stigma in adults towards children with MHPs, and perhaps in the children towards themselves, therefore removing barriers to further help‐seeking [45].

There was strong rejection (e.g. high levels of ‘unimportant’ or ‘should not be included’) from all three panels pertaining to approaches that involved the first aider talking about their own mental health or challenging life experiences, and panellists provided comments that explicitly stated that the first aider should avoid doing this (Table 3). Certain statements also produced wide variance in the panels’ rating of their importance, particularly amongst the lived experience experts (Table 4). The statements that were rated higher for lived experience experts, but lower for the other two panels, centred around acknowledging the commonness of MHPs, persisting with communicating with the child, even if the conversation is a little uncomfortable, and allowing the child to talk to a mental health professional alone. Generally, lived experience experts were more concerned with helping the child, even if they were reluctant, whilst the educators appeared to be less convinced about allowing children to speak to mental health professionals alone or the first aider asking other adults close to the child to monitor their mental health. A greater percentage of health professionals (82.1% vs. 65.5% of lived experience and 56.1% of educators) rated asking others to monitor the child as being important to providing mental health first aid for children, which may reflect health professionals' understanding of the importance of holistic support for children.

There was also contention around what language to use when describing MHPs with children, with none of the statements about using or not using mental health diagnostic labels (e.g. ‘depression’, ‘anxiety’) being endorsed. Instead, the final recommendation was to use language appropriate to the developmental stage of the child and reflecting the child's own use of language. This may reflect some hesitation around what appropriate language would be to use with children when discussing MHPs. Other research has suggested that adults tend to use lay mental health terminology when talking to or about their own children (such as stress or worry) but diagnostic terms when talking about outgroups or strangers (such as anxiety), which may perpetuate mental health stigma [38]. Using child‐developed language around mental health may help to integrate outgroup judgements to allow adults to more openly communicate with children about mental health.

Issues of confidentiality raised many comments from the panels during the first round of the Delphi study, particularly for the teacher and health professional panels, who hold obligations with regard to confidentiality and mandatory reporting. The final endorsed statements around never promising the keep the conversation a secret may reflect this high professional consideration for the safety of children and reflects the role that many first aiders may have to navigate as professionals, mandatory reporters and first aiders.

### 4.1 Limitations

This study had some limitations that should be noted. Although we recruited a large sample from a variety of countries, drop‐off in panellist numbers occurred across rounds. This may have been due to the size of the survey (over 300 items) and the demands on participants' time. Although the retention between rounds was above 70%, from Round 1 to Round 3 the retention rate was just above 40%. Nevertheless, this study used a sample of experts from a variety of countries worldwide, with a diversity of types of expertise, and the rigorous process of the Delphi method means we can be confident that the strategies recommended here constitute safe, effective mental health first aid for supporting children.

### 4.2 Implications and Future Research

The guidelines developed by this Delphi study are based on the consensus of expert stakeholders and the first resource to outline the knowledge and skills that adults require when supporting children with a mental health problem. These guidelines are relevant for developing training and education programs for members of the public. The guidelines developed in this study may only be relevant to those in high‐income, Westernized countries, therefore, future research should seek to replicate the design using panellists selected from other countries that have different health care systems and cultures. Further research could also explore how these guidelines might be implemented in practice, develop training that can build key skills in assisting children, and evaluate whether this training is effective in improving adults' mental health literacy for supporting children and mental health and wellbeing outcomes for children themselves.

## 5 Conclusion

The guidelines developed in this expert consensus study offer a framework for approaching a conversation with a child whether the adult is a parent, teacher or health professional. As well as being a resource for adult supporters of children, they will be useful to inform the content of training of adults in a child's support network to effectively intervene when MHPs begin to emerge.

## Author Contributions

**Catherine L. Johnson:** conceptualisation, data curation, formal analysis; methodology, investigation, project administration, writing–original draft, writing–review and editing. **Claire M. Kelly:** methodology, investigation, supervision, writing–review and editing. **Anthony F. Jorm:** methodology, investigation, supervision, funding acquisition, writing–review and editing. **William Garvey:** supervision, writing–review and editing, validation. **Laura M. Hart:** conceptualisation, methodology, investigation, supervision, funding acquisition, project administration, writing–review and editing, writing–original draft. All authors read and approved the final version of this manuscript.

## Ethics Statement

Ethics approval for this study was received from the University of Melbourne (Project ID: 21342). All participants provided active consent.

## Conflicts of Interest

L.M.H. is a volunteer director of the health promotion charity The Embrace Collective. C.M.K. is an employee of Mental Health First Aid International. A.F.J. is a nonexecutive Director of Mental Health First Aid International. None of the authors stand to receive any incentive, financial or otherwise, from the publication of this paper.

## Supporting information

Supporting information.

Supporting information.

## Data Availability

The data that support the findings of this study are available on request from the corresponding author. The data are not publicly available due to privacy or ethical restrictions.

## References

[1] L. Godoy , N. D. Mian , A. S. Eisenhower , and A. S. Carter , “Pathways to Service Receipt: Modeling Parent Help‐Seeking for Childhood Mental Health Problems,” Administration and Policy in Mental Health and Mental Health Services Research 41, no. 4 (2014): 469–479, 10.1007/s10488-013-0484-6. 23504296 PMC3740083

[2] S. E. Teagle , “Parental Problem Recognition and Child Mental Health Service Use,” Mental Health Services Research 4, no. 4 (2002): 257–266, 10.1023/A:1020981019342. 12558014

[3] A. R. Stiffman , B. Pescosolido , and L. J. Cabassa , “Building a Model to Understand Youth Service Access: The Gateway Provider Model,” Mental Health Services Research 6, no. 4 (2004): 189–198, 10.1023/B:MHSR.0000044745.09952.33. 15588030 PMC3745273

[4] N. R. Koning , F. L. Büchner , M. E. A. Verbiest , R. R. J. M. Vermeiren , M. E. Numans , and M. R. Crone , “Factors Associated With the Identification of Child Mental Health Problems in Primary Care—A Systematic Review,” European Journal of General Practice 25, no. 3 (2019): 116–127, 10.1080/13814788.2019.1623199. 31246106 PMC6713156

[5] K. Sayal and E. Taylor , “Detection of Child Mental Health Disorders by General Practitioners,” The British journal of General Practice: The Journal of the Royal College of General Practitioners 54, no. 502 (2004): 348–352. 15113517 PMC1266168

[6] T. Reardon , K. Harvey , M. Baranowska , D. O'brien , L. Smith , and C. Creswell , “What Do Parents Perceive Are the Barriers and Facilitators to Accessing Psychological Treatment for Mental Health Problems in Children and Adolescents? A Systematic Review of Qualitative and Quantitative Studies,” European Child & Adolescent Psychiatry 26, no. 6 (2017): 623–647, 10.1007/s00787-016-0930-6. 28054223 PMC5446558

[7] L. M. Hart , A. F. Jorm , C. L. Johnson , et al., “Mental Health Literacy for Supporting Children: The Need for a New Field of Research and Intervention,” World psychiatry : Official Journal of the World Psychiatric Association (WPA) 22, no. 2 (2023): 338–339, 10.1002/wps.21099. 37159366 PMC10168171

[8] C. M. Kelly , A. F. Jorm , and A. Wright , “Improving Mental Health Literacy as a Strategy to Facilitate Early Intervention for Mental Disorders,” Medical Journal of Australia 187, no. S7 (2007): 26–30, 10.5694/j.1326-5377.2007.tb01332.x. 17908021

[9] L. A. Tully , D. J. Hawes , F. L. Doyle , M. G. Sawyer , and M. R. Dadds , “A National Child Mental Health Literacy Initiative Is Needed to Reduce Childhood Mental Health Disorders,” Australian and New Zealand Journal of Psychiatry 53, no. 4 (2019): 286–290, 10.1177/0004867418821440. 30654614

[10] L. M. Hart , C. M. Kelly , and A. F. Jorm , Supporting Child Mental Health: How Adults Can Provide Mental Health First Aid for Children Aged 5–12 years (Independently published, 2024).

[11] B. A. Kitchener , A. F. Jorm , and C. M. Kelly , Mental Health First Aid Manual (Mental Health First Aid Australia, 2013).

[12] L. M. Hart , R. J. Mason , C. M. Kelly , S. Cvetkovski , and A. F. Jorm , “Teen Mental Health First Aid’: A Description of the Program and an Initial Evaluation,” International Journal of Mental Health Systems 10, no. 1 (2016): 3, 10.1186/s13033-016-0034-1. 26788123 PMC4717562

[13] C. M. Kelly , J. M. Mithen , J. A. Fischer , et al., “Youth Mental Health First Aid: A Description of the Program and an Initial Evaluation | International Journal of Mental Health Systems,” International Journal of Mental Health Systems 5, no. 4 (2011): 4, https://link.springer.com/article/10.1186/1752-4458-5-4. 21272345 10.1186/1752-4458-5-4 PMC3041764

[14] N. Kendall‐Taylor , 2009, Conflicting Models of Mind in Mind: Mapping the Gaps Between the Expert and the Public Understandings of Child Mental Health as Part of Strategic Frame AnalysisTM, The FrameWorks Institute, https://www.frameworksinstitute.org/wp-content/uploads/2020/03/childmentalhealthculturalmodels.pdf.

[15] N. Kendall‐Taylor and A. Mikulak . 2009, Child Mental Health: A Review of the Scientific Discourse, A FrameWorks Research Report, The FrameWorks Institute, https://www.frameworksinstitute.org/wp-content/uploads/2020/06/childmentalhealthreview.pdf.

[16] B. A. Pescosolido , P. S. Jensen , J. K. Martin , B. L. Perry , S. Olafsdottir , and D. Fettes , “Public Knowledge and Assessment of Child Mental Health Problems: Findings From the National Stigma Study‐Children,” Journal of the American Academy of Child and Adolescent Psychiatry 47, no. 3 (2008): 339–349, 10.1097/CHI.0b013e318160e3a0. 18216729

[17] D. Hurley , C. Swann , M. S. Allen , H. L. Ferguson , and S. A. Vella , “A Systematic Review of Parent and Caregiver Mental Health Literacy,” Community Mental Health Journal 56, no. 1 (2020): 2–21, 10.1007/s10597-019-00454-0. 31541315

[18] B. Mathews , “Mandatory Reporting Laws and the Identification of Severe Child Abuse and Neglect,” in Mandatory Reporting Laws: Their Origin, Nature, and Development Over Time, eds. B. Mathews and D. C. Bross (Netherlands: Springer, 2015), 3–25, 10.1007/978-94-017-9685-9_1.

[19] B. Mathews and M. C. Kenny , “Mandatory Reporting Legislation in the United States, Canada, and Australia: A Cross‐Jurisdictional Review of Key Features, Differences, and Issues,” Child Maltreatment 13, no. 1 (2008): 50–63, 10.1177/1077559507310613. 18174348

[20] C. L. Johnson , M. A. Gross , A. F. Jorm , and L. M. Hart , “Mental Health Literacy for Supporting Children: A Systematic Review of Teacher and Parent/Carer Knowledge and Recognition of Mental Health Problems in Childhood,” Clinical Child and Family Psychology Review 26, no. 3 (2023): 569–591, 10.1007/s10567-023-00426-7. 36763174 PMC10123050

[21] K. Buhagier and J. Cassar , “Common Mental Health Disorders in Children and Adolescents in Primary Care: A Survey of Knowledge, Skills and Attitudes Among General Practitioners in a Newly Developed European Country,” European Journal of Psychiatry 26, no. 3 (2012): 145–158, 10.4321/S0213-61632012000300001.

[22] T. Lempp , M. Heinzel‐Gutenbrunner , and C. Bachmann , “Child and Adolescent Psychiatry: Which Knowledge and Skills Do Primary Care Physicians Need to Have? A Survey in General Practitioners and Paediatricians,” European Child & Adolescent Psychiatry 25, no. 4 (2016): 443–451, 10.1007/s00787-015-0757-6. 26250895

[23] A. R. Miller , C. Johnston , A. F. Klassen , S. Fine , and M. Papsdorf , “Family Physicians' Involvement and Self‐Reported Comfort and Skill in Care of Children With Behavioral and Emotional Problems: A Population‐Based Survey,” BMC Family Practice 6, no. 1 (2005): 12, 10.1186/1471-2296-6-12. 15762982 PMC1079811

[24] C. M. Kerns , D. L. Robins , P. T. Shattuck , C. J. Newschaffer , and S. J. Berkowitz , “Expert Consensus Regarding Indicators of a Traumatic Reaction in Autistic Youth: A Delphi Survey,” Journal of Child Psychology and Psychiatry 64, no. 1 (2023): 50–58, 10.1111/jcpp.13666. 35817758 PMC10368297

[25] M. K. Murphy , N. A. Black , D. L. Lamping , et al., “Consensus Development Methods, and Their Use in Clinical Guideline Developmen,” Health Technology Assessment 2, no. 3 (1998): 1–88, 10.3310/hta2030. 9561895

[26] S. Jünger , S. A. Payne , J. Brine , L. Radbruch , and S. G. Brearley , “Guidance on Conducting and REporting DElphi Studies (CREDES) in Palliative Care: Recommendations Based on a Methodological Systematic Review,” Palliative Medicine 31, no. 8 (2017): 684–706, 10.1177/0269216317690685. 28190381

[27] A. F. Jorm , “Using the Delphi Expert Consensus Method in Mental Health Research,” Australian & New Zealand Journal of Psychiatry 49, no. 10 (2015): 887–897, 10.1177/0004867415600891. 26296368

[28] J. A. Fischer , C. M. Kelly , B. A. Kitchener , and A. F. Jorm , “Development of Guidelines for Adults on How to Communicate With Adolescents About Mental Health Problems and Other Sensitive Topics: A Delphi Study,” Sage Open 3, no. 4 (2013): 2158244013516769, 10.1177/2158244013516769.

[29] A. M. Ross , L. M. Hart , A. F. Jorm , C. M. Kelly , and B. A. Kitchener , “Development of Key Messages for Adolescents on Providing Basic Mental Health First Aid to Peers: A Delphi Consensus Study,” Early Intervention in Psychiatry 6, no.3 (2012): 229–238, 10.1111/j.1751-7893.2011.00331.x. 22240091

[30] W. T. Gattrell , P. Logullo , E. J. van Zuuren , et al., “ACCORD (ACcurate COnsensus Reporting Document): A Reporting Guideline for Consensus Methods in Biomedicine Developed via a Modified Delphi,” PLoS Medicine 21, no. 1 (2024): e1004326, 10.1371/journal.pmed.1004326. 38261576 PMC10805282

[31] C. M. Kelly , A. F. Jorm , and B. A. Kitchener , “Development of Mental Health First Aid Guidelines on How a Member of the Public Can Support a Person Affected by a Traumatic Event: A Delphi Study,” BMC Psychiatry 10, no. 1 (2010): 49, 10.1186/1471-244X-10-49. 20565918 PMC2904289

[32] C. M. Kelly , A. F. Jorm , B. A. Kitchener , and R. L. Langlands , “Development of Mental Health First Aid Guidelines for Suicidal Ideation and Behaviour: A Delphi Study,” BMC Psychiatry 8, no. 1 (2008): 17, 10.1186/1471-244X-8-17. 18366657 PMC2324091

[33] A. M. Manyara , A. Purvis , O. Ciani , G. S. Collins , and R. S. Taylor , “Sample Size in Multistakeholder Delphi Surveys: At What Minimum Sample Size Do Replicability of Results Stabilize?,” Journal of Clinical Epidemiology 174 (2024): 111485, 10.1016/j.jclinepi.2024.111485. 39069013 PMC7617918

[34] A. M. Ross , C. M. Kelly , and A. F. Jorm , “Re‐Development of Mental Health First Aid Guidelines for Non‐Suicidal Self‐Injury: A Delphi Study,” BMC Psychiatry 14, no. 1 (2014): 236, 10.1186/s12888-014-0236-5. 25134432 PMC4197339

[35] M. B. H. Yap , P. D. Pilkington , S. M. Ryan , C. M. Kelly , and A. F. Jorm , “Parenting Strategies for Reducing the Risk of Adolescent Depression and Anxiety Disorders: A Delphi Consensus Study,” Journal of Affective Disorders 156 (2014): 67–75, 10.1016/j.jad.2013.11.017. 24359862

[36] R. Boulkedid , H. Abdoul , M. Loustau , O. Sibony , and C. Alberti , “Using and Reporting the Delphi Method for Selecting Healthcare Quality Indicators: A Systematic Review,” PLoS One 6, no. 6 (2011): e20476, 10.1371/journal.pone.0020476. 21694759 PMC3111406

[37] S. Unigwe , C. Buckley , L. Crane , L. Kenny , A. Remington , and E. Pellicano , “GPs' Confidence in Caring for Their Patients on the Autism Spectrum: An Online Self‐Report Study,” British Journal of General Practice 67, no. 659 (2017): e445–e452, 10.3399/bjgp17X690449. PMC5442960 28483821

[38] J. Mueller , M. M. Callanan , and K. Greenwood , “Parents' Communication to Primary School‐Aged Children About Mental Health and Ill‐Health: A Grounded Theory Study,” Journal of Public Mental Health 13, no. 1 (2014): 13–19, 10.1108/JPMH-09-2013-0063.

[39] U. Bronfenbrenner and P. A. Morris , “The Bioecological Model of Human Development,” in Handbook of Child Psychology: Theoretical Models of Human Development, Vol. I, 6th ed. (John Wiley & Sons, 2007).

[40] S. Kutcher , Y. Wei , and C. Coniglio , “Mental Health Literacy: Past, Present, and Future,” Canadian Journal of Psychiatry 61, no. 3 (2016): 154–158, 10.1177/0706743715616609. 27254090 PMC4813415

[41] National Mental Health Commission ,National Children's Mental Health and Wellbeing Strategy. (Australian Government, 2020), https://consultation.mentalhealthcommission.gov.au/policy-projects/childrens-mental-health-and-wellbeing-strategy/.

[42] C. C. Chang , Y. M. Chen , T. L. Liu , R. C. Hsiao , W. J. Chou , and C. F. Yen , “Affiliate Stigma and Related Factors in Family Caregivers of Children With Attention‐Deficit/Hyperactivity Disorder,” International Journal of Environmental Research and Public Health 17, no. 2 (2020): 576, 10.3390/ijerph17020576. 31963190 PMC7013698

[43] N. S. Salleh , K. L. Abdullah , T. L. Yoong , S. Jayanath , and M. Husain , “Parents' Experiences of Affiliate Stigma When Caring for a Child With Autism Spectrum Disorder (ASD): A Meta‐Synthesis of Qualitative Studies,” Journal of Pediatric Nursing 55 (2020): 174–183, 10.1016/j.pedn.2020.09.002. 32957021

[44] P. W. Corrigan and A. C. Watson , “How Children Stigmatize People With Mental Illness,” International Journal of Social Psychiatry 53, no. 6 (2007): 526–546, 10.1177/0020764007078359. 18181355

[45] A. J. Morgan , A. Ross , and N. J. Reavley , “Systematic Review and Meta‐Analysis of Mental Health First Aid Training: Effects on Knowledge, Stigma, and Helping Behaviour,” PLoS One 13, no. 5 (2018): e0197102, 10.1371/journal.pone.0197102. 29851974 PMC5979014

