# Supplementary material for: How to Approach a Child About Concerns for Their Mental Health and Seeking Help: A Delphi Expert Consensus Study to Develop Guidelines on Mental Health First Aid for Supporting Children
Source: Health Expect. 2025 Jan 13;28(1):e70126. doi: 10.1111/hex.70126 (PMC11729744; doi:10.1111/hex.70126)
Supplement: Supplementary file 1 — Supporting information. [file HEX-28-e70126-s001.docx]

**Supplementary File A**

**Search terms**

**Academic literature**

These search terms were used are part of a previous study on the mental health literacy of parents and educators who live and work with children (Johnson et al., 2023).

| **Search Term** | **APA Index (PsychINFO)** | **MeSH heading (MEDLINE)** | **Thesaurus search (ERIC)** |
| --- | --- | --- | --- |
| **Literacy** |  |  |  |
| Literacy | Mental Health Literacy | Health Literacy | N/A |
| Stigma | Stigma  Mental Health Stigma | Stigma, social | [Labeling (of Persons)](javascript:XslPostBack('ctl00$ctl00$MainContentArea$MainContentArea$xslResults','ThesaurusLink','LinkTarget%7CauthorityDetail%24LinkTerm%7CSU%2B%2522Labeling%2B%2528of%2BPersons%2529%2522%24ResultID%7C1');) |
| Attitude | Parental Attitudes  Adult Attitudes  Teacher Attitudes  Community Attitudes  Health Personnel Attitudes  Mental Illness (Attitudes Toward) | Attitude | [Beliefs](javascript:XslPostBack('ctl00$ctl00$MainContentArea$MainContentArea$xslResults','ThesaurusLink','LinkTarget%7CauthorityDetail%24LinkTerm%7CDE%2B%2522Beliefs%2522');) |
| Attitude | Parental Attitudes  Adult Attitudes  Teacher Attitudes  Community Attitudes  Health Personnel Attitudes  Mental Illness (Attitudes Toward) | Attitude | [Attitudes](javascript:XslPostBack('ctl00$ctl00$MainContentArea$MainContentArea$xslResults','ThesaurusLink','LinkTarget%7CauthorityDetail%24LinkTerm%7CDE%2B%2522Attitudes%2522');)+ |
| Perception | Perception | N/A | [Perception](javascript:XslPostBack('ctl00$ctl00$MainContentArea$MainContentArea$xslResults','ThesaurusLink','LinkTarget%7CauthorityList%24LinkTerm%7CDE%2B%2522Perception%2522');) |
| Competence | Competence |  | [Competence](javascript:XslPostBack('ctl00$ctl00$MainContentArea$MainContentArea$xslResults','ThesaurusLink','LinkTarget%7CauthorityList%24LinkTerm%7CDE%2B%2522Competence%2522');) |
| Understanding | Comprehension | Comprehension | [Comprehension](javascript:XslPostBack('ctl00$ctl00$MainContentArea$MainContentArea$xslResults','ThesaurusLink','LinkTarget%7CauthorityDetail%24LinkTerm%7CSU%2B%2522Comprehension%2522%24ResultID%7C0');) |
| Ability | Ability | Aptitude | Ability |
| Knowledge | Health Knowledge  Knowledge (General) | Knowledge | NA |
| Identification | Identification |  | Identification |
| Awareness | Awareness | Awareness | [Perception](javascript:XslPostBack('ctl00$ctl00$MainContentArea$MainContentArea$xslResults','ThesaurusLink','LinkTarget%7CauthorityList%24LinkTerm%7CDE%2B%2522Perception%2522%24ResultID%7C0');) |
| Recognition | Recognition (Learning) | Recognition, Psychology | Recognition (psychology) |
| Label | Labeling |  | Labeling (of persons) |
| Referral | Professional referral |  | Referral |
| Help seeking | Help seeking behaviour  Health Care seeking behaviours | Help seeking behaviour | Help seeking |
| (Mental health literacy or belief* or attitude* or perception* or stigma or competenc* or abilit* or capabilit* or confiden* or know* or identif* or aware* or recogni* or label* or referral* or help-seeking).mp. | exp Mental health literacy/ or exp Stigma/ or exp Mental Health Stigma/ or exp Parental Attitudes/ or exp Adult Attitudes/ or exp Teacher Attitudes/ or exp Community Attitudes/ or exp Health Personnel Attitudes/ or exp Mental Illness/ or exp Perception/ or exp competence/ or exp ability/or exp Health Knowledge/ or exp Knowledge/or exp Identification/ or exp Awareness/ or exp Recognition/ or exp Labeling/ or exp Professional Referral/ or exp help seeking behaviour/ or exp health care seeking behaviours/ | exp Health literacy/ or exp stigma, social/ or exp attitude/ or exp comprehension/ or exp aptitude/ or exp knowledge/ or exp awareness/ or exp recognition, psychology/ or exp help seeking behavior/.mh | (((((((((DE "Labeling (of Persons)") OR (DE "Beliefs")) OR (DE "Attitudes")) OR (DE "Perception")) OR (DE "Comprehension")) OR (DE "Ability")) OR (DE "Identification")) OR (DE "Recognition (Psychology)")) OR (DE "Referral")) OR (DE "Help Seeking") |
| **Mental illness** |  |  |  |
| Mental health | Mental Health | Mental Health | Mental Health |
| Mental illness | Mental disorders | Mental disorders | Mental disorders |
| Depression | Major depression  Depression (Emotion) | Depression  Depressive Disorders | Depression (psychology) |
| Anxiety | Anxiety  Anxiety Disorders  Generalized Anxiety Disorder  Separation Anxiety  Separation Anxiety Disorder  Social Anxiety | Anxiety  Anxiety Disorders  Anxiety, Separation  Phobia, Social | Anxiety  Anxiety disorders |
| Mood Disorder | Affective disorders | Mood disorders  Affective symptoms  Affective disorders | NA |
| Disruptive Behaviour | Behaviour Problems  Behaviour Disorders  Disruptive Behaviour Disorders  Classroom Behaviour  Aggressive Behaviour  Classroom Behaviour Modification  Classroom discipline | Attention Deficit and Disruptive Behavior Disorders  Conduct disorders  Antisocial Personality Disorder | Antisocial behavior  Behavior disorders  Behavior problems |
| Eating Disorder | Eating disorders | Feeding and eating disorders  *Narrowers:* Feeding and eating disorders of childhood | Eating disorders |
| Internalizing |  | No equivalent | NA |
| Externalizing | Attention Deficit Disorder with Hyperactivity  Conduct Disorder  Oppositional Defiant Disorder  Autism Spectrum Disorders | No equivalent | NA |
| (Mental health or mental illness or mental disorder* or depress* or anxi* or mood disorder or affective disorder or disruptive behav* or eating disorder or internali* or externali*).mp. | exp Mental Health/ or exp Mental disorders/ or exp Major depression/  or exp Anxiety/  or exp Anxiety Disorders/  or exp Generalized Anxiety Disorder/  or exp Separation Anxiety/  or exp Separation Anxiety Disorder/  or exp Social Anxiety/ or exp Affective disorders/ or exp Behavior Problems/ or  exp Behavior Disorders/ or  exp Disruptive Behavior Disorders/ or  exp Classroom Behavior/ or  exp Aggressive Behavior/ or  exp Classroom Behavior Modification/ or  exp Classroom discipline/ or  exp Eating disorders/ or exp Attention Deficit Disorder with Hyperactivity/ or  exp Conduct Disorder/ or  exp Oppositional Defiant Disorder/ or  exp Autism Spectrum Disorders/ | exp Mental health/ or exp mental disorders/ or exp depression/ or exp depressive disorders/ or exp anxiety/ or exp anxiety disorders/ or exp anxiety, separation/ or exp phobia, social/ or exp mood disorders/ or exp affective symptoms/ or exp affective disorders/ or exp conduct disorders/ or exp antisocial personality disorder/.mh | (((((((DE "Mental Health") OR (DE "Mental Disorders")) OR (DE "Depression (Psychology)")) AND (DE "Anxiety" OR DE "Anxiety Disorders")) OR (DE "Antisocial Behavior")) OR (DE "Behavior Disorders")) OR (DE "Behavior Problems")) OR (DE "Eating Disorders") |
| **Population** |  |  |  |
| Children | No equivalent term | Child  Child, preschool | Children |
| Student | Elementary school students  Middle school students  Primary school students | Students | Elementary schools  Elementary education |
| Teacher | Elementary school teachers  Middle school teachers  Teachers | School teachers  *Narrowers:* Elementary School Teachers  Pre-school Teachers | Elementary school teachers |
| Parent | Parents | Parents | Parents |
| Family | Family | Family | Family (sociological unit) |
| Mother | Mothers | Mothers (human and animal) | Mothers |
| Father | Fathers | Fathers (human and animal) | Fathers |
| Caregivers | Caregivers | Caregivers  *Narrowers:* Family caregivers | Caregivers |
| Guardian | Guardianship | Legal Guardians | NA |
| Gatekeeper | No equivalent term | Gatekeeping  ~~Gatekeeper, Health Services~~ | NA |
| (primary school teacher* or elementary school teacher* or grade school teacher* or middle school teacher* or Parent* or Mother or Father or Famil* or Caregiv*; or guardian* or gatekeeper* or gate-keeper* or gate-keeper*).mp. | exp Elementary school teachers/ or  exp Middle school teachers/ or  exp Teachers/ or exp Parents/ or exp Family/ or exp Mothers/ or exp Fathers/ or exp Caregivers/ or exp Guardianship/ | exp Elementary school teachers/ or exp parents/ or exp family/ or exp mothers/ or exp fathers/ or exp legal guardians/ or exp caregivers/.mh | (((((DE "Elementary School Teachers") OR (DE "Family (Sociological Unit)")) OR (DE "Parents")) OR (DE "Mothers")) OR (DE "Fathers")) OR (DE "Caregivers") |
| (primary school* or junior school* or elementary school* or grade school*).mp. | exp Elementary school students/ or exp  Middle school students/ or exp  Primary school students/ | exp Child/.mh |  |
