# Supplementary material for: How to Approach a Child About Concerns for Their Mental Health and Seeking Help: A Delphi Expert Consensus Study to Develop Guidelines on Mental Health First Aid for Supporting Children
Source: Health Expect. 2025 Jan 13;28(1):e70126. doi: 10.1111/hex.70126 (PMC11729744; doi:10.1111/hex.70126)
Supplement: Supplementary file 2 — Supporting information. [file HEX-28-e70126-s002.docx]

**Supplementary File B**

Finalized guidelines

**Mental Health First Aid for Supporting Children:**

Guidelines on how to talk to a child aged 5-12 years about their mental health and seeking help

**Definition of mental health first aid for children**

Mental health first aid for children is defined as the help that is given to support a child aged 5-12 years who (1) is developing a mental health problem or is experiencing a worsening of an existing problem, (2) has experienced an adverse life event that places them at increased risk of poor mental health, or (3) is in a mental health crisis. The first aid is given to the child or their primary support system until appropriate help is received.

**Background knowledge that a mental health first aider should have**

The more knowledge that you have about child mental health, the more confident you may feel in approaching the child to discuss your concerns. Having sufficient knowledge about normal child development and behaviours will also help you to recognise when a child is developing a mental health problem.

Adults providing mental health first aid to a child should have a basic understanding of the following.

***How to recognise mental health problems*in children**

This includes the common mental health problems diagnosed in childhood and the signs of these. You should also understand how mental health problems may present differently in children with neurodevelopmental disorders.

***Risk and protective factors for children***

You should know the risk factors for mental health problems in children, including the impact of trauma. Knowledge of the protective factors for good child mental health can also be drawn on when providing first aid.

***Services available for child mental health***

This includes which professionals can make diagnoses and treat mental health problems in children, as well as the referral pathways and local services available for children. It is also useful to know about the range of appropriate resources that are available to help adults who support children with mental health problems.

**Preparing to give mental health first aid**

Before talking to the child, think about your approach, as well as where and when you might have a conversation with the child.

***Tailor your approach to the child***

When preparing to provide mental health first aid, you should consider the age, maturity and needs of the child. Be aware that children differ in their cognitive and emotional development and that you may need to tailor your approach, language and actions accordingly. You should also consider the warning signs and risk factors relevant to the child, e.g. history of trauma. However, do not make assumptions about what is causing a child's mental health problem or what is happening in the child's life.

***Consider the best way to communicate with the child***

It is best to use language and communication strategies that are appropriate to the child's age and developmental level. Some children may have difficulty communicating about their mental health, especially if they have additional needs or disabilities. You should offer these children more time and support when discussing mental health.

Remember that children may not always have the language to communicate how they feel. Be aware that when children are struggling with their emotions, they may find it harder than usual to communicate how they are feeling.

***Consider who could help***

You may want to consider who the best person is to provide mental health first aid to the child. For more information about how to speak to other adults who are involved in caring for the child, about your concerns for the child’s mental health, please see the *Guidelines on how to speak to other adults about your concerns for a child’s mental health*.

***Find a time and place***

If you are concerned about a child’s mental health, try to talk to the child as soon as possible, but not at the expense of ensuring a comfortable situation for the conversation. Choose a time and place to talk with the child where you both feel comfortable and where nobody will disturb you. If age and developmentally appropriate, try to involve the child in any decisions about when and where to have the conversation.

Try to create an emotionally safe place where the child can talk openly. Ask the child if they would like to have another trusted adult present.

***Consider the child’s family and community context***

It is important to consider the child's mental health problems within the context of the child's family, school and community. If you are from a different culture to the child, you should try to find out about how mental health problems are understood in the child's culture. Be careful to show respect for the child's family at all times.

***Consider your own view of mental health***

Be mindful of any negative attitudes you might have towards mental illness, as the child may pick up on this. A child’s fear of being judged or treated differently can stop them from opening up during a conversation about their mental health.

**Talking to the child about their mental health**

***Create a supportive atmosphere***

Early in the conversation, try to build trust and rapport with the child, being guided by their interests, skills and strengths. Tell them you care about them.

Be guided by the child’s reactions or responses in determining the direction and pace of the conversation, or when deciding whether to continue the conversation, move onto another topic, or give the child some space to settle. Slow down or pause the conversation if the child becomes distressed or disengaged. Let the child know it's okay to ask for a break if they need one.

Reassure the child that they can tell you anything they feel comfortable talking about, that you will take the time to answer any questions they have, and that you won't get angry or upset with them.

Be aware that you may need to have more than one conversation about mental health with the child in order for them to share openly with you.

***Confidentiality***

At the start of the conversation, you should explain to the child that there are limits to what can be kept confidential (e.g. “kept between us”), and when and why other adults may need to be informed. Never promise to keep the conversation a secret and be clear with the child about who you will tell any information that they provide. If age and developmentally appropriate, try to work with the child to decide who else should be informed.

When discussing confidentiality, check that the child has understood what has been said in an age- and developmentally-appropriate way, e.g. by asking the child to reflect back to you in the their own words what they understand.

***Talk about your concerns***

Talk to the child about what you have noticed that makes you concerned about them and encourage them to discuss any concerns they have, e.g. “Is there anything you wanted to talk about? You haven't really seemed like yourself lately”. Using open questions and a warm and caring communication style can help encourage the child to talk. Try to communicate in a calm and kind way, particularly if the child is anxious.

***Listen actively***

Give the child your full attention. Show them that you are actively listening, for example, by using reflective statements. In addition to listening to the child's words, you should also observe the child's tone of voice and body language. Ask follow-up questions of the child to show your own understanding.

***Think about your body language and tone of voice***

Try to keep your body language open and relaxed. You might find it useful to sit down at the child's level. Try to stay calm when speaking with the child, and avoid arguments, confrontation or hostility.

***Resist the urge to problem-solve***

Be aware that the child might prefer to talk and be heard, rather than focus on problem-solving. Avoid immediately jumping into trying to solve any problem they might bring up and instead let them know that you are there to listen.

***Encourage questions***

During the conversation, give the child time to think and ask questions. You can encourage them by suggesting they ask questions whenever they want.

Try to answer the child's questions honestly and directly, taking into account the age, maturity and needs of the child. If you do not know the answer to a question the child asks, be honest and tell the child you don't know but will find out the answer.

***Take their concerns seriously***

Take the child's concerns seriously, even if they don't feel like a big deal to you. Do not use words or language that minimises or invalidates the child's problem. For example, do not label the child as “silly” or “attention-seeking”. Do not promise the child that everything will be fine.

If the child has fears, do not ignore, minimise or make fun of these, even if they appear trivial to you. Accept the child's feelings, even if they are different to your feelings. If the child has shared their feelings, praise them for this.

***Empathise***

Try to think about the situation from the child's point of view by putting yourself in the child's shoes and showing empathy for their situation. Try to remain non-judgemental. However, if the child expresses myths or misconceptions about mental health problems, try to correct these, e.g. boys shouldn't cry, girls shouldn't get angry.

Tell them that they are not to blame for any feelings they might be having.

***Explain mental health***

If you give the child information about mental health, keep it simple and relevant so that they can understand. Explain that it is common to experience problems with mental health and that there are ways to deal with them. If they are describing physical symptoms (e.g. stomach-ache, headache), explain how sometimes thoughts and feelings can lead to physical symptoms. Making the connection between emotional feelings and physical sensations in an age- and developmentally-appropriate way can be helpful when talking about mental health problems with children.

Reassure the child that there is help available. It can be useful to have information about helplines and online chat services for the child in case they are needed.

Throughout the conversation, check that the child has understood what has been said in an age and developmentally appropriate way, e.g. by asking the child to reflect back to you in the child's own words what they understand. Pause regularly when delivering new information to allow the child time to process.

Do not use negative or stigmatising language, e.g. weak, crazy, naughty.

***Avoid reacting negatively***

Do not react too strongly to what the child has to say. Do not express negative judgement about the child or their situation; or shame, criticise or blame the child.

**Handling difficulties in the conversations**

***If the child is reluctant to talk to you***

Do not take it personally if the child does not open up straight away.

Tell the child that you are here to talk if they would like to. Ask them if there is another trusted adult they would be willing to talk to and reassure them that it is okay to talk to another adult about what is concerning them. Then seek support from other adults who could help the child.

If the child does not like to talk, or is having difficulty communicating, use other ways to help them communicate, e.g. notes, drawing, modelling, playing with toys. Consider whether the child might find it easier to talk while doing another activity, such as playing a game, drawing, or kicking a ball.

***If the child has a strong emotional reaction to the conversation***

If the child has an emotional reaction to the conversation, acknowledge this and use accurate emotional language, e.g. angry, sad, scared. Reassure the child that they are not going to get in trouble with you for talking about how they feel, and that expressing feelings is okay.

***When unsure of what to do next***

If you can't figure out what to do next in helping the child, seek help from another trusted adult who could provide a new perspective on the child's problem. For more information about how to speak to other adults who are involved in caring for the child, about your concerns for the child’s mental health, please see the *Guidelines on how to speak to other adults about your concerns for a child’s mental health*.

**Seeking Help**

Remember that as a first aider, you should not attempt to diagnose a child's mental health problem.

***Encourage early help***

You should be aware that the earlier a child receives appropriate professional help, the better the outcome is likely to be, and thus it is appropriate to advocate for the child to receive appropriate professional help as soon as possible. Explain to the child that because of the concerns that you have, you are going to talk to someone who can help.

***Knowing when the child needs professional help***

You should seek professional help for the child if:

- You believe there is risk of harm to the child or others
- You notice symptoms that last longer than two weeks or are having a major impact on the child's life (sleeping, eating, schooling, peer relationships, family relationships)
- The child shows a high level of distress.

***What is appropriate help for a child?***

You should be aware that appropriate help for a child with a mental health problem should ideally involve parents, teachers and health professionals working together to support the child. Try to identify one or two adults in the child's life who can keep an eye on how the child is going, e.g. a parent may ask a teacher, or a health professional may ask a parent. Discuss with the child who they feel most comfortable talking to about their problems. Offer them the option of talking to other trusted adults about their problem and encourage the child to talk to them as well.

For older children (approximately 9 to 12 years old), try to work with the child to come up with a plan together for what to do next and who else to involve. Offer to revisit the plan if the child's situation changes or if the child wishes to re-engage with the conversation.

Try to enlist the help of parents when supporting the child, unless doing so would put the child at risk. If you are not the child’s parent, you should talk to the child about how they would like their parents to support them. You might also want to recommend good quality programs and resources (e.g. online programs for parents) to other adults in the child’s life, if they are not aware of them.

If you are a teacher and you have ongoing concerns about the child or you are worried about the child's reaction to the conversation, seek advice from a mental health professional or an appropriate school staff member with wellbeing responsibility, such as a social worker or school counsellor.

***If the child is reluctant to get others involved***

If the child is reluctant for you to talk to others about their mental health, you should explain why you have to talk to another adult, then give the child time to think about who they would like to have help from.

If the child is reluctant to seek help, you should ask the child about their worries and concerns. If you are a parent or a health professional, you might explain to the child that they need help, and you will not ignore or avoid the problem. If you are a parent, and the child is concerned about the process of seeking help, you should look at appropriate resources with the child, e.g. picture books, short videos about going to the doctor or counsellor.

For an older child, you might ask them whether they would like to speak to a health professional alone when they attend an appointment.

***If the child is at risk of harm***

There are separate guidelines for assisting a child who is experiencing a mental health crisis, or a child who has experienced a traumatic or adverse life event.

If the child reports any of the following, please follow the appropriate guidelines:

- Self-harm, suicidal thoughts or feelings, or expressing a wish to die
- Abuse or trauma

***Encourage coping strategies***

While waiting for professional help, encourage the child to try to use coping strategies that are evidence-based, e.g. relaxation techniques. In order to suggest strategies that may be useful for the child's self-care, you can ask the child what makes them feel happy or what they enjoy. Also encourage other adults who care for the child to use evidence‐based strategies (e.g. good sleep habits, regular physical activity, relaxation techniques) to support the child's mental health.

-----------------------------------------------------------------------------------------------------------

**About these Guidelines**

***Purpose of these guidelines***

These guidelines are designed to help members of the public, and especially parents, teachers and health professionals, to provide mental health first aid to a child who is developing a mental health problem. This document focuses on how to speak to a child directly about your concerns for their mental health and about seeking help.

This is one of a set of guidelines about how to support children aged 5-12 years with mental health problems. The other guidelines are:

- How to speak to other adults about your concerns for a child’s mental health
- How to support a child who is at risk of suicide or self-harm
- How to support a child who is threatening violence or displaying aggression
- How to support a child who has experienced an adverse or traumatic event

***Development of these guidelines***

These guidelines were developed using the Delphi expert consensus method. This involved gathering three different kinds of experts and asking them to agree on which first aid strategies were the most appropriate for adults supporting a child who is developing a mental health problem. Experts included (1) parents who have cared for a child with a mental health problem, or young adults who experienced a mental health problem in their earlier school years, (2) teachers who have expertise in supporting children with mental health problems, and (3) health professionals who provide clinical services to children with mental health problems. Experts were from Australia, Germany, Ireland, New Zealand, Switzerland and the USA.

These guidelines were developed through a collaboration between researchers at the University of Melbourne and Mental Health First Aid International. Funding for this project was provided by a National Health and Medical Research Council grant provided to Anthony Jorm and by funding from the North West Melbourne Primary Health Network awarded to Laura Hart.

***How to use these guidelines***

It is important to tailor your support to the needs of the child you are helping. These guidelines are a general set of recommendations only. They are most suitable for providing mental health first aid in high-income countries with developed health systems.

Although these guidelines are copyright, they can be freely reproduced for non-profit purposes provided the source is acknowledged. Please cite these guidelines as follows: Johnson, C., Kelly, C., Jorm, A., Garvey, W. & Hart, L. (2024) *Mental Health First Aid for Supporting Children: Guidelines on how to talk to a child aged 5-12 years about their mental health and seeking help.* Self-published: Melbourne. They can be accessed at <https://mspgh.unimelb.edu.au/centres-institutes/centre-for-health-equity/research-group/equity-and-mental-health>

Enquiries should be sent to [lhart@unimelb.edu.au](mailto:lhart@unimelb.edu.au) or [mhfasc-research@unimelb.edu.au](mailto:mhfasc-research@unimelb.edu.au)
